# Contrast-enhanced ultrasound for the characterization of portal vein thrombosis vs tumor-in-vein in HCC patients: a systematic review and meta-analysis

**DOI:** 10.1007/s00330-019-06649-z

**Published:** 2020-02-04

**Authors:** Jifan Chen, Jianing Zhu, Chao Zhang, Yue Song, Pintong Huang

**Affiliations:** Department of Ultrasound, The Second Affiliated Hospital, Zhejiang University School of Medicine, Hangzhou, Zhejiang China

**Keywords:** Contrast media, Ultrasonography, Portal vein, Hepatocellular carcinoma

## Abstract

**Objectives:**

Portal vein thrombosis (PVT) is a common complication of liver cirrhosis. However, differentiation of thrombosis and tumor-in-vein (TIV) may be challenging. Contrast-enhanced ultrasound (CEUS) is an excellent method for detection of vascularization and could help in the distinction. We performed a systematic review and meta-analysis for evaluating the diagnostic value of CEUS in differentiating between PVT and TIV in hepatocellular carcinoma (HCC) patients.

**Methods:**

PubMed, Embase, Cochrane Library, and Web of Science were searched up to the 5th of May 2019. The study quality was assessed by QUADAS-2 tool. Pooled sensitivity and specificity were calculated by the bivariate random effect model and hierarchical summary receiver-operating characteristic (SROC) curve was plotted.

**Results:**

Seven studies including 425 participants were analyzed after screening 986 articles searched from databases. The pooled sensitivity and specificity of CEUS in diagnosing TIV were 0.94 (95%CI, 0.89–0.97) and 0.99 (95%CI, 0.80–1.00), respectively. The area under the curve (AUC) of SROC curve was 0.97 (95%CI, 0.95–0.98). The pooled sensitivity and AUC were consistent across all the subgroups of different subject numbers, country, study design, CEUS contrast agents, and diagnostic criteria.

**Conclusions:**

CEUS is highly efficient in differentiating TIV from PVT and is an alternative or a substitute for CT and/or MRI.

**Trial registration:**

PROSPERO registration number: CRD42019138847

**Key Points:**

*• Characterization of portal vein thrombosis (PVT) vs tumor-in-vein (TIV) is critical for HCC staging.*

*• CEUS has an excellent safety profile, provides a real-time analysis without any loss in accuracy compared with CT and MRI.*

*• This meta-analysis demonstrates that contrast-enhanced ultrasound (CEUS) is a suitable method for the detection of PVT and distinction with TIV.*

**Electronic supplementary material:**

The online version of this article (10.1007/s00330-019-06649-z) contains supplementary material, which is available to authorized users.

## Introduction

Portal vein thrombosis (PVT), which is the formation of a thrombus within the portal vein trunk and intrahepatic portal branches, is a common complication of liver cirrhosis [1–3]. PVT prevalence in cirrhotic patients (≥ 26%) increases in advanced liver diseases, such as hepatocellular carcinoma (HCC) and intrahepatic cholangiocarcinoma (ICC) [4]. Owing to portal hypertension caused by sclerosis of the hepatic lobule or compression by tumor, slow-flowing blood coagulates and forms either partial or complete PVT. Advanced HCC commonly invades the portal vein. In such cases, both PVT and tumor-in-vein (TIV) could be associated. In China, TIV incidence ranged from 44 to 62.2% in HCC patients concomitant with PVT [5]. The presence of TIV is a factor of poorer prognosis and affects treatment strategy. HCC patients without TIV were defined as Barcelona-Clínic Liver Cancer (BCLC) 0/A/B, considering related symptoms, liver function, tumor size, and number. HCC patients with TIV are not eligible for resection or liver transplantation. Median survival rate drops down from more than 60 months to less than 11 months [6–9]. They are candidates for sorafenib targeted therapy.

B-mode or color Doppler ultrasound has an excellent value for detecting HCC, as well as PVT [10, 11]. It is recommended (AASLD 2018 Practice Guideline) biannually in cirrhosis patients as a screening tool for HCC [12]. Although some attempts were made to characterize TIV with ultrasound, the accuracy is not optimal [13, 14]. Several studies have confirmed that Liver Imaging Reporting and Data System of CEUS (CEUS LI-RADS) enables standardized diagnosis of HCC with substantial diagnostic efficiency [15, 16]. One study reported good potential of CEUS LI-RADS with modified score system for diagnosing HCC and ICC [17]. Besides, one meta-analysis reported a high sensitivity and specificity for CEUS in HCC diagnosis, which were 0.85 (95%CI, 0.84–0.86) and 0.91 (95%CI, 0.90–0.92), respectively [18]. In addition, the EASL and WFUMB-EFSUMB guidelines mentioned CEUS as a method to distinguish between tumor and thrombosis in portal vein especially in patients with underlying HCC, high level of serum alpha-fetoprotein, and enlarged portal vein diameter [10, 19]. Thrombosis is avascular and non-enhanced in the arterial phase, whereas TIV involve malignant vascularity, which is enhanced in the arterial phase with sign of washout in the portal and late phases.

In the recent 15 years, efforts in differentiating PVT and TIV using CEUS were made and the results were inspiring. Although reviews describing the diagnostic value of CEUS for TIV have emerged recently [20, 21], there are no diagnostic systematic review and meta-analysis regarding CEUS proficiency in TIV diagnosis. We aimed to systematically evaluate the already published original articles, combine all the reliable evidence, and assess the application of CEUS in distinction of TIV from PVT.

## Methods

### Search strategies

This study was performed following the guideline of the Cochrane Handbook for Systematic Reviews of Diagnostic Test Accuracy. We searched four databases, i.e., PubMed, Embase, Cochrane Library, and Web of Science, for articles published up to the 5th of May 2019. The search strategies were the combination of MeSH terms, entry terms (synonyms), study keywords, and search filters for diagnostic tests [22]. The search terms included (“portal vein thromb*” OR “portal vein embolus” OR “PVT” OR (“tumor in vein” AND “portal vein”)) AND (“contrast enhanced ultraso*” OR “CEUS” OR “contrast enhanced sonography” OR “contrast-enhanced ultraso*” OR “contrast-enhanced doppler ultrasonography”).

### Study selection

Two reviewers (Chen and Zhu) independently screened titles and abstracts, and discrepancies were eliminated via discussion. The articles were carefully reviewed with the following eligibility criteria:

1. Diagnostic studies with retrospective or prospective design that applied CEUS in detecting TIV.
2. Reference standard for the differentiation between PVT and TIV should be specified in the study.
3. Studies should report the patients’ hepatic medical history clearly and report the concrete number of true-positive (TP), true-negative (TN), false-positive (FP), and false-negative (FN) or diagnostic accuracy parameters such as sensitivity and specificity to construct two × two contingency tables for CEUS in diagnosing between TIV and PVT.
4. Full-text should be written in English.
5. Studies were excluded if they included duplicate data, or if some patients might overlap among studies; as such, only the studies with comprehensive patients’ information were used.

### Data extraction and quality assessment

Two reviewers (Chen and Zhu) independently extracted the data and discrepancy was eliminated by discussion. Study characteristics, patients’ features, and data of diagnostic results (e.g., TP, TN, FP, FN, sensitivity, and specificity) of each included study were extracted.

Two other reviewers (Zhang and Song) independently assessed the quality of each study using the Quality Assessment of Diagnostic Accuracy Studies-2 (QUADAS-2) tool and disagreement was resolved by discussion.

### Statistical analysis

Data was analyzed using the Stata 15.0 software (Midas commands) (StataCorp LP, College Centre) and Review Manager (RevMan) 5.3 (The Nordic Cochrane Centre, The Cochrane Collaboration).

For each study, extracted information was used to construct two × two contingency tables. The bivariate random effect model based on the binomial distribution for sensitivity and specificity was performed. To determine whether a threshold effect is present, we applied the spearman correlation analysis with *p* < 0.05 representing a threshold effect. The pooled sensitivity and specificity were displayed with pooled point estimate and 95% confidence intervals (CIs). The hierarchical summary receiver-operating characteristic (ROC) curve was plotted and the area under the curve (AUC) was calculated. The Fagan plot was performed to estimate how much probability that a patient suffers from TIV would change by CEUS [23].

The *I*^2^ index was calculated to estimate the heterogeneity, which shows the total variation (in percentage) across studies due to heterogeneity rather than chance. The overall studies demonstrated substantial heterogeneity if the *I*^2^ index value is greater than 50% and vice versa. Covariates that may contribute to heterogeneity were evaluated by subgroup analysis to figure out the origin of heterogeneity.

Subgroup analysis was conducted in consideration of study scale in regard to enrolled subject numbers, country, study design (prospective or retrospective), blind method, specific CEUS contrast agents, and diagnostic criteria if based on pathology. Sensitivity analysis was also performed to address quality differences and validate the robustness of our result.

To investigate the publication bias, Deeks’ funnel plot with a regression line was plotted [24]. The slope coefficient is suggestive of an asymmetric funnel plot if *p* < 0.10 [25].

## Results

### Search results

The initial search from four databases identified 986 articles, of which 312 were removed because of duplication and 560 were removed after reading the title and abstract carefully. Besides, 80 articles were excluded due to article type, of which eight were reviews and 72 were conference abstracts. Twenty non-English language articles were removed from the screen list of full-text assessment.

**Fig. 1:**
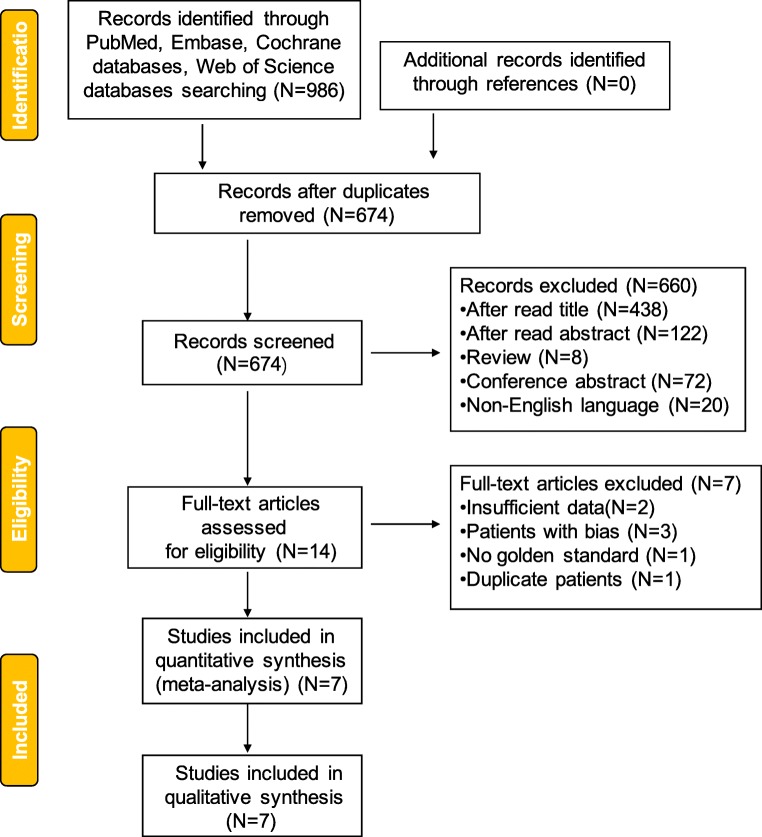
Flowchart of selection of studies for inclusion in the meta-analysis

After reading fourteen articles in the full-text assessment list, two of them failed to provide sufficient data, three of them enrolled unsuitable patients, who might cause bias if enrolled, and one had no reference standard. One was removed due to duplicate patients. Finally, seven articles were included in this systematic review and meta-analysis (Fig. 1, Table 1).

**Table 1:** Feature of included studies

Author, year	Country	Predesign	Number of participants	TIV/PVT	Age	Gender (male/female)	Medical history	Reference standard	Contrast agents
Norio Ueno, 2006	Japan	Prospective, but blind not mentioned	55	40/15	66 (53–83)	43/12	Chronic liver diseases	Pathology, CT or angiography follow-up	Levovist
Paolo Sorrentino, 2009	Italy	Prospective, blind	108	58/50	66 ± 6	82/26	Cirrhosis with HCC	Pathology (biopsy) and follow-up	SonoVue
Ze-Zhou Song, 2010	China	Prospective, blind	17	14/3	38–78	14/3	Cirrhosis with HCC	Pathology and follow-up	SonoVue
Sandro Rossi, 2008	Italy	Prospective, blind	50	44/6	67 ± 5	39/11	Cirrhosis with HCC	Pathology	SonoVue
Paolo Sorrentino, 2011	Italy	Prospective, blind	96	72/24	66 (43–88)	61/35	Cirrhosis with HCC	Pathology	SonoVue
Maria C Chammas, 2019	Brazil	Prospective, blind	43	22/21	64 (51–77)	31/12	Chronic liver diseases and HCC-suggestive nodules	> 6 months imaging follow-up	PESDA, Definity, or SonoVue
P. RiCCI, 2000	Italy	Not mentioned	56	16/40	57 (47–79)	39/17	Cirrhosis	Pathology and CT/MRI imaging	Levovist

*TIV*, tumor-in-vein; *PVT*, portal vein thrombosis; *HCC*, hepatocellular carcinoma

### Evaluation of study quality by QUADAS-2

**Fig. 2:**
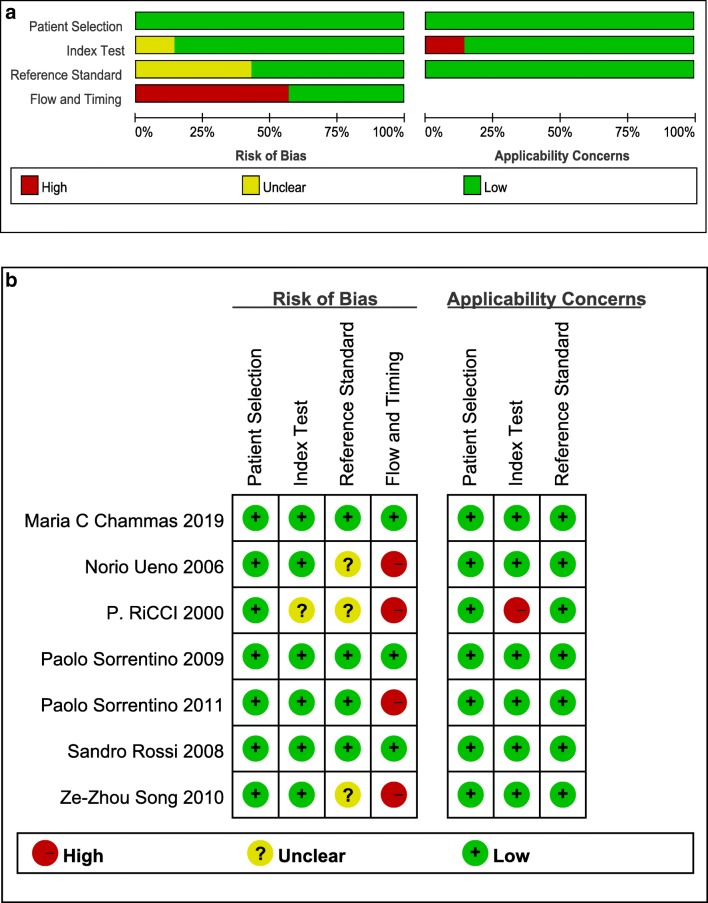
Risk of bias and applicability concerns. **a** Risk of bias and applicability concerns summary. **b** Risk of bias and applicability concerns graph

QUADAS-2 was selected as the tool for evaluating the included studies. This consists of two parts to assess the study quality: the risk of bias part contains four categories (patient selection, index test, reference standard, and flow and timing) and the applicability concerns part contains three categories (patient selection, index test, and reference standard). The risk grades were assigned as high, unclear, and low by two independent reviewers (Zhu and Song) and the discrepancies were settled via discussion. All seven included studies demonstrated a low risk of bias and no applicability concerns in patient selection (Fig. 2). One study [26] has an unclear risk of bias and high applicability concern in index test, because contrast-enhanced color Doppler imaging was adopted rather than contrast-enhanced ultrasound imaging to characterize TIV from PVT, which might cause bias and limit the applicability. About 42.8% of the studies [26–28] were graded as unclear risk of bias in reference standard because these studies did not report whether the application of reference standard was in a blind feature. Some studies applied pathological results as the only reference standard, while some applied a mixture of pathology and imaging follow-up; therefore, 58.2% (4/7) of the studies [26–29] were graded as high risk of bias in the category of flow and timing. In summary, using the QUADAS-2 tool, the included studies have a low concern regarding applicability in all the three categories and low risk of bias in patient selection and index test categories, but might be at risk in the categories of reference standard and flow and timing.

### Diagnostic value of CEUS in the characterization of TIV from PVT

**Fig. 3:**
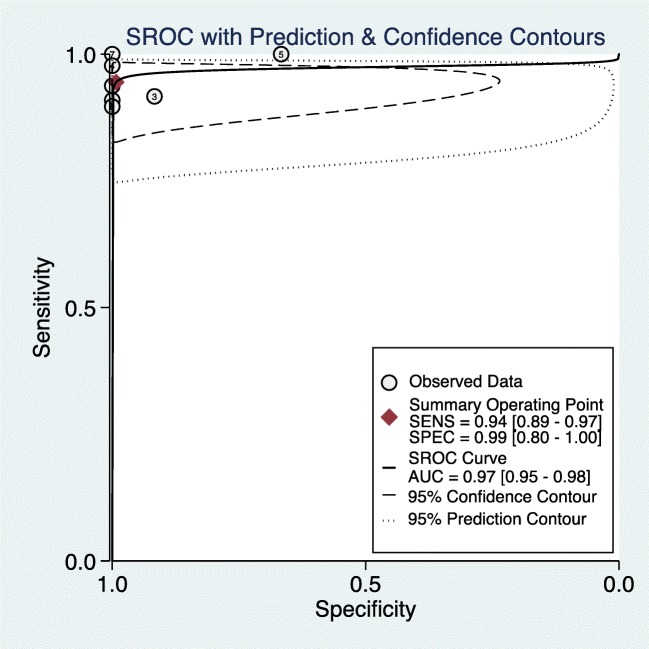
Summary receiver-operating characteristic (SROC) curve. Bivariate random effect model based on the binomial distribution for sensitivity and specificity

Among the seven included studies, no threshold effect was detected (*p* = 0.590). The summary sensitivity and specificity of CEUS in diagnosing TIV were 0.94 (95%CI, 0.89–0.97) and 0.99 (95%CI, 0.80–1.00) respectively. After plotting the summary ROC (SROC) curve, the calculated value of AUC of SROC was 0.97 (95%CI, 0.95–0.98) (Fig. 3, supplementary Table 1).

**Fig. 4:**
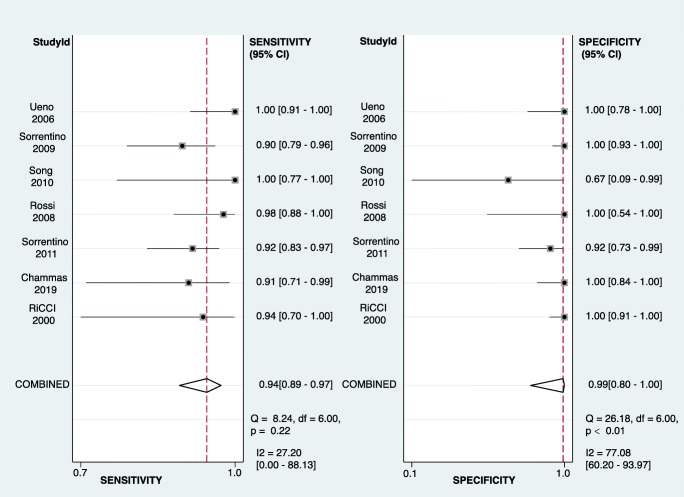
Forest plot of sensitivity and specificity among studies. Levels of significance: *p < 0.05 (Bivariate random effect model based on the binomial distribution for sensitivity and specificity)

According to the forest plot (Fig. 4), the *I*^2^ of summary sensitivity and specificity is 27.2% (*p* = 0.22) and 77.08% (*p* < 0.01). One study [28] showed obvious heterogeneity from the others in specificity, because only 3 patients without TIV were enrolled in this study.

### Subgroup analysis and sensitivity analysis

Due to the limited number of false-positive subjects (three in total) in our review, some subgroup analysis and sensitivity analysis results were difficult to obtain due to the convergence problem. The sensitivity and AUC were consistent across all the subgroups, whereas the 95%CI of the specificity within subgroups of study scale, country, and diagnostic criteria varied a lot when compared with the overall result (Table 2). Except for Song’s study [28] with a lower 95%CI of specificity of 0.51, the sensitivity analysis indicated little quality difference among the included studies (supplementary Table 2).

**Table 2:** Subgroup analysis results

Subgroup	Population	Study number	Number of participants	Sensitivity (95%CI)	Specificity (95%CI)	AUC (95%CI)
All combined	Overall	7	425	0.94 (0.89, 0.97)	0.99 (0.80, 1.00)	0.97 (0.95, 0.98)
Subject number	≥ 50	5	365	0.95 (0.89, 0.98)	1.00 (0.45, 1.00)	0.98 (0.90, 1.00)
	< 50	2	60	–	–	–
Country	Italy	4	310	0.93 (0.88, 0.96)	0.99 (0.65, 1.00)	0.93 (0.84, 0.98)
	Non-Italy	3	115	–	–	–
Predesign	Prospective	6	369	0.95 (0.88, 0.98)	0.98 (0.79, 1.00)	0.98 (0.88, 1.00)
	Retrospective	1	56	–	–	–
	Blind	5	314	0.93 (0.88, 0.96)	0.98 (0.79, 1.00)	0.96 (0.85, 0.99)
	Non-blind	2	111	–	–	–
Diagnostic criteria	Pathological only	4	259	0.93 (0.86, 0.97)	0.97 (0.10, 1.00)	0.94 (0.87, 0.97)
Contrast agents	SonoVue	4	271	0.94 (0.88, 0.97)	0.96 (0.76, 0.99)	0.96 (0.90, 0.99)
	Non-SonoVue	3	154	–	–	–

### Publication bias

**Fig. 5:**
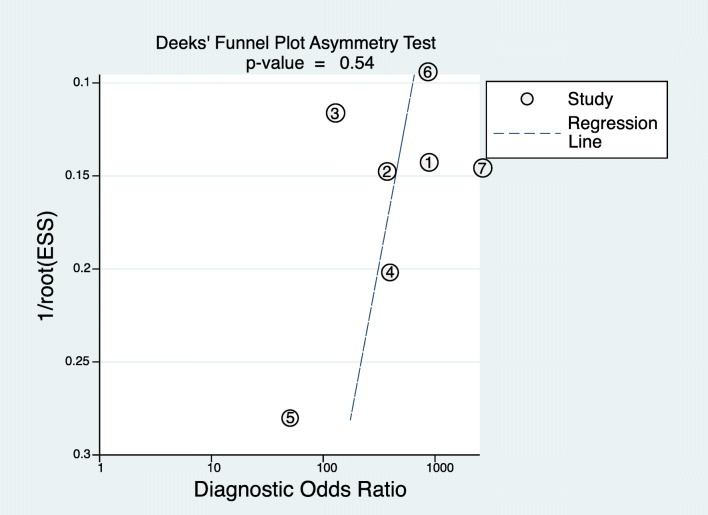
Funnel plot of publication bias among studies. Levels of significance: *p < 0.10 (Deeks’ funnel plot asymmetry test)

Publication bias was not detected by performing Deeks’ funnel plot in this study. Besides, the slope coefficient indicated no significant small sample size bias (Fig. 5, *p* = 0.54).

### Posterior probability of CEUS in characterizing TIV

The Fagan plot (supplementary Fig. 1) demonstrated that CEUS is very informative in raising the probability of diagnosing TIV from 50 to 99% when positive and lowering the probability of malignancy to as low as 5% when negative.

## Discussion

This study is the first meta-analysis that summarizes CEUS studies for the diagnosis of TIV in HCC patients, concomitant with PVT. According to the Oxford 2011 Levels of Evidence (OCEBM levels) [30], studies enrolled in our meta-analysis provided level 2 or level 3 evidence in evidence-based medicine [31]. As depicted in the results, with low to moderate risk of bias and low applicability concern, CEUS embraces excellent diagnostic accuracy with pooled sensitivity and pooled specificity of 0.94 (95%CI, 0.89–0.97) and 0.99 (95%CI, 0.80–1.00), respectively. This demonstrated that CEUS is an ideal modality for portal vein evaluation in suspected or proved HCC patients. The diagnostic efficiency assessed by AUC remained consistently high when the studies applied different contrast agents, study design, diagnostic criteria, study scales, and countries. Since the specificity of the diagnostic test is calculated based on true-negative and false-positive subjects, in this study, the number of subjects with a negative condition according to the reference standard was 159, but only three of them were false-positives, which might result in the heterogeneity in reported specificity. Combining the results of *I*^2^ and sensitivity analysis, the overall heterogeneity was acceptable.

PVT could easily be detected by B-mode ultrasound with a hyperechoic mass in the lumen of the dilated portal vein. Color Doppler ultrasound is the first choice of imaging modality for detecting PVT and visualizes the flow within the portal vein and demonstrates high sensitivity and specificity [32]. Regarding TIV, B-mode ultrasound might be helpless if the lesion is discontinuous with HCC. The probability of malignancy increases when color Doppler ultrasound detects pulsatile arterial flow within the PVT [33]; still, the sensitivity is low [13]. The EASL/AASLD extension criteria for non-invasive portal vein lesion characterization indicated the necessity for biopsy rather than relying on imaging techniques [29]. However, portal vein biopsy can be difficult or non-productive.

In clinical practice, PVT patients with suspected or proven HCC usually undergo contrast-enhanced CT or MRI to get a one-time overall assessment of the primary lesions (mostly in the liver) and the secondary lesions such as TIV, PVT, bile duct occlusion, and nearby invasion. Dual-energy CT with iodine quantification is a useful tool to distinguish between TIV and PVT (AUC = 0.993, sensitivity = 100%, specificity = 95.2%) [34]. Three-dimensional reconstruction of multiple-slice computed tomography is helpful [35]. Besides, published studies have reported high diagnostic accuracy (up to 95%) for differentiating malignant component from benign PVT by gadoxetic acid–enhanced MR imaging [36]. Susceptibility-weighted MRI was superior to diffusion-weighted MR imaging in distinguishing the malignant component in portal vein with a high diagnostic capability (AUC, 0.989; sensitivity, 95%; specificity, 95.5%) [37–40].

In our department, CEUS is performed simultaneously with routine US in case of tumor, in order to gain rapid and reliable information. CEUS is usually performed prior to CT or MRI scan in most cases because CEUS owns the advantages of being convenient, cheap, real-time, and non-irradiative with comparable diagnostic performance. The accuracy, sensitivity, and specificity are consistent between CEUS and contrast CT in diagnosing and classifying malignant PVT [41]. Interestingly, CEUS appears to be significantly superior to CT for the detection and characterization of TIV in HCC in one study [42]. CEUS could also provide the quantitative analysis parameters to interpret the perfusion flow within portal vein lesions. Some drawbacks might affect the diagnostic performance of CEUS imaging. Firstly, the operator dependency compared with other contrast imaging methods might cause variation in the actual diagnostic performance by user’s proficiency and experience. Secondly, due to the potentially compromised ultrasound access by abdominal gas, CEUS may not be able to detect the extension to other splanchnic vessels, unlike CT or MRI. Overweight and image artifacts might also affect the results. Up until now, the comparison of diagnostic values for TIV across different contrast-enhanced imaging technologies (CEUS, CE-CT, and CE-MRI) remains unsettled. Due to the limited number of studies, the network meta-analysis for quantitatively comparing the diagnostic values among the above imaging technologies could not be implemented.

Some limitations exist in our meta-analysis. Firstly, a limited number of studies were included in this meta-analysis due to the limited relevant high-quality studies. Secondly, the reference standard was not consistent among studies and the time interval between CEUS imaging examination and the standard reference was vague. It was reported that the average growth velocity of portal vein tumor thrombus is 0.9 ± 1.0 mm/day in HCC patients. Based on the rapid progression of TIV, defining an appropriate interval between CEUS imaging and standard reference becomes important [43]. In our review, the assessment of flow and timing in QUADAS-2 tool was at risk, and further studies should take this interval with caution to get more convincing results.

In conclusion, this comprehensive meta-analysis demonstrated the performance of CEUS in the characterization of TIV from PVT. Owing to the advantages and diagnostic efficiency mentioned above, CEUS would earn a place in distinguishing between TIV and PVT in the future. The comparative studies focusing on the benefit and diagnostic accuracy of different imaging modalities in distinguishing TIV should be carried out.

## Electronic supplementary material

ESM 1 (DOCX 249 kb)

## Abbreviations

AUC: Area under the curve
BCLC: Barcelona Clínic Liver Cancer
CEUS: Contrast-enhanced ultrasound
CEUS LI-RADS: The Liver Imaging Reporting and Data System of CEUS
CI: Confidence intervals
FN: False negative
FP: False positive
HCC: Hepatocellular carcinoma
ICC: Intrahepatic cholangiocarcinoma
PVT: Portal vein thrombosis
QUADAS-2: Quality assessment of diagnostic accuracy studies-2
SROC: Summary receiver-operating characteristic
TIV: Tumor-in-vein
TN: True negative
TP: True positive
