# Supplementary material for: Contrast-enhanced ultrasound for the characterization of portal vein thrombosis vs tumor-in-vein in HCC patients: a systematic review and meta-analysis
Source: Eur Radiol. 2020 Feb 4;30(5):2871–80. doi: 10.1007/s00330-019-06649-z (PMC7160216; doi:10.1007/s00330-019-06649-z)
Supplement: Supplementary file 1 — (DOCX 249 kb) [file 330_2019_6649_MOESM1_ESM.docx]

**Supplementary data**

**Contrast-enhanced ultrasound for the characterization of portal vein thrombosis vs Tumor-in-Vein in HCC patients: a systematic review and meta-analysis**

**Table of contents:**

**Supplementary Table 1** Data extract from included studies

**Supplementary Table 2** Sensitivity analysis results

**Supplementary Fig. 1** Fagan plot

| **Supplementary Table 1** Data extract from included studies | | | | | | | | | |
| --- | --- | --- | --- | --- | --- | --- | --- | --- | --- |
| Author, year | TP | FP | FN | TN | Accuracy (%) | Sensitivity (%) | Specificity (%) | PPV (%) | NPV (%) |
| Norio Ueno, 2006 | 40 | 0 | 0 | 15 | 100 | 100 | 100 | 100 | 100 |
| Paolo Sorrentino, 2009 | 52 | 0 | 6 | 50 | 94.40 | 89.60 | 100 | 100 | 89.00 |
| Ze-Zhou Song, 2010 | 14 | 1 | 0 | 2 | 94.12 | 100 | 66.70 | 93.00 | 100 |
| Sandro Rossi, 2008 | 43 | 0 | 1 | 6 | 98.00 | 98.00 | 100 | 100 | 86.00 |
| Paolo Sorrentino, 2011 | 66 | 2 | 6 | 22 | 91.60 | 91.60 | 91.60 | 97.00 | 79.00 |
| Maria C Chammas, 2019 | 20 | 0 | 2 | 21 | 95.00 | 91.00 | 100 | 100 | 91.00 |
| P. RiCCI, 2000 | 15 | 0 | 1 | 40 | 98.20 | 94.00 | 100 | 100 | 98.00 |
| *TP*, true positive; *FP*, false positive; *FN*, false negative; *TN*, true negative; *PPV*, positive predictive value; *NPV*, negative predictive value | | | | | | | | | |

| **Supplementary Table 2** Sensitivity analysis results | | | |
| --- | --- | --- | --- |
| Excluded study | Sensitivity (95%CI) | Specificity (95%CI) | AUC (95%CI) |
| Non | 0.94 (0.89, 0.97) | 0.99 (0.80, 1.00) | 0.97 (0.95, 0.98) |
| Norio Ueno, 2006 | 0.94 (0.89, 0.97) | 0.99 (0.80, 1.00) | 0.97 (0.89, 0.99) |
| Paolo Sorrentino, 2009 | 0.96 (0.91, 0.98) | 0.99 (0.80, 1.00) | 0.98 (0.91, 1.00) |
| Ze-Zhou Song, 2010 | 0.94 (0.89, 0.97) | 1.00 (0.51, 1.00) | 0.97 (0.89, 0.99) |
| Sandro Rossi, 2008 | 0.93 (0.89, 0.96) | 0.99 (0.81, 1.00) | 0.94 (0.85, 0.98) |
| Paolo Sorrentino, 2011 | -- | -- | -- |
| Maria C Chammas, 2019 | 0.95 (0.88, 0.98) | 0.99 (0.79, 1.00) | 0.98 (0.90, 1.00) |
| P. RiCCI, 2000 | 0.95 (0.88, 0.98) | 0.98 (0.79, 1.00) | 0.98 (0.88, 1.00) |
|  | | | |

**
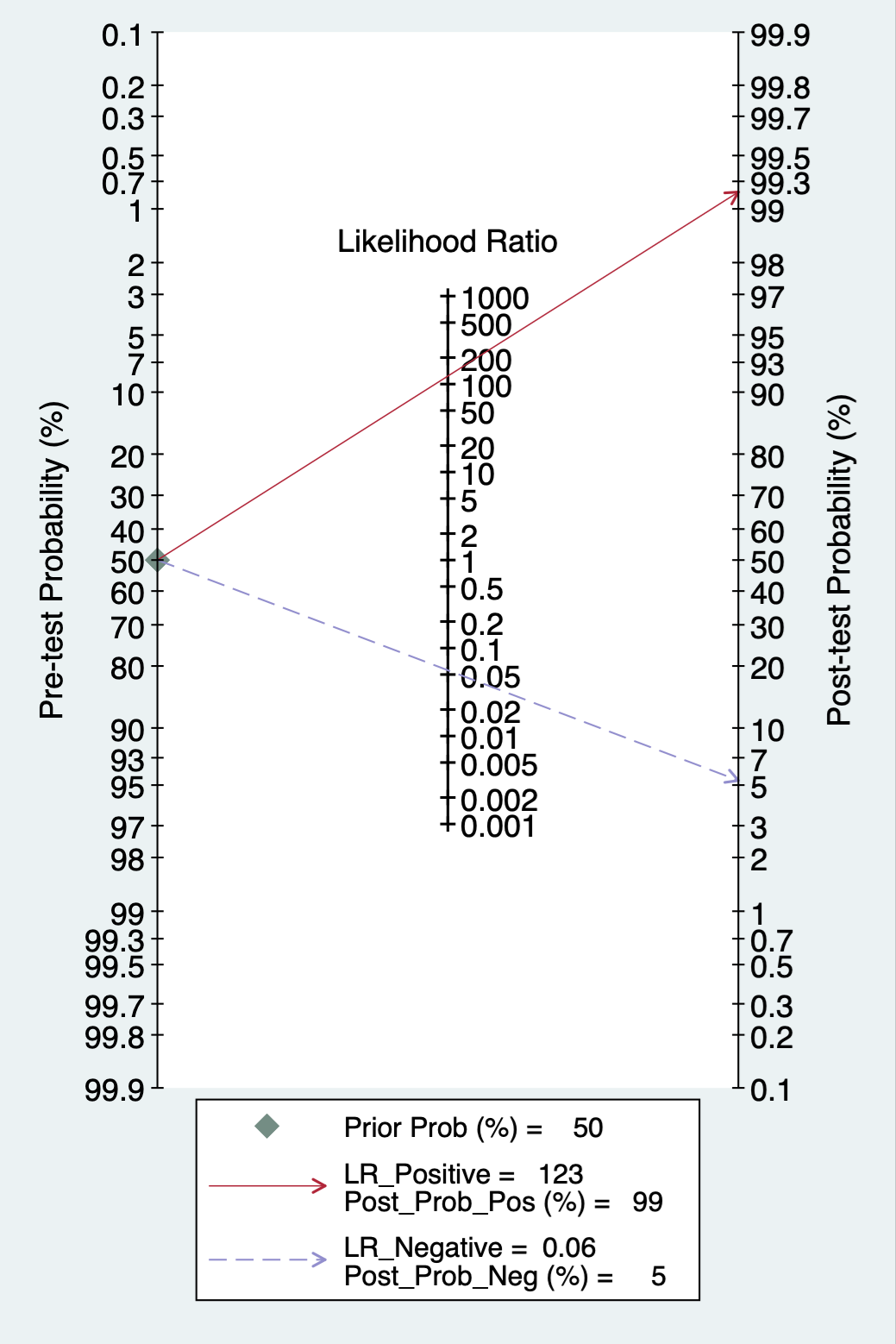
**

**Supplementary Fig. 1** Fagan plot

*LR*, likelihood ratio
